# DBN Structure Design Algorithm for Different Datasets Based on Information Entropy and Reconstruction Error

**DOI:** 10.3390/e20120927

**Published:** 2018-12-04

**Authors:** Jianjun Jiang, Jing Zhang, Lijia Zhang, Xiaomin Ran, Jun Jiang, Yifan Wu

**Affiliations:** National Digital Switching System Engineering and Technological Research Center (NDSC), Zhengzhou 450000, Henan, China

**Keywords:** deep learning, DBN, artificial intelligence, structure design, information entropy, reconstruction error, improved simulated annealing algorithm

## Abstract

Deep belief networks (DBNs) of deep learning technology have been successfully used in many fields. However, the structure of a DBN is difficult to design for different datasets. Hence, a DBN structure design algorithm based on information entropy and reconstruction error is proposed. Unlike previous algorithms, we innovatively combine network depth and node number and optimizes them simultaneously. First, the mathematical model of the structural design problem is established, and the boundary constraint for node number based on information entropy is derived by introducing the idea of information compression. Moreover, the optimization objective of the network performance based on reconstruction error is proposed by deriving the fact that network energy is proportional to reconstruction error. Finally, the improved simulated annealing (ISA) algorithm is used to adjust the DBN network layers and nodes simultaneously. Experiments were carried out on three public datasets (MNIST, Cifar-10 and Cifar-100). The results show that the proposed algorithm can design its proper structure to different datasets, yielding a trained DBN which has the lowest reconstruction error and prediction error rate. The proposed algorithm is shown to have the best performance compared with other algorithms and can be used to assist the setting of DBN structural parameters for different datasets.

## 1. Introduction

A deep belief network (DBN) is a kind of deep artificial neural network (ANN) [1]. An ANN, which originated from Rosenblatt’s perceptron model, is an information processing network composed of simple nodes that has nonlinear fitting ability [2]. In 2006 and later, Hinton proposed the DBN [3] and CD-K [4] algorithms, which has enabled ANNs to develop from a shallow to deep structure, achieving significant performance improvements. As a typical type of deep network [5], DBNs are widely used in image processing [6,7,8,9,10], speech recognition [11,12,13] and nonlinear function prediction [14], yielding excellent performance. However, DBNs still have many problems worth studying, such as the network structure design [15,16,17,18,19], selection and improvement of training algorithms [20,21], introduction of automatic encoders, and implementation of GPU parallel acceleration [22,23]. In particular, the design of DBN network structures is of high research significance.

The performance of a DBN is closely related to its structure. A simple structure can improve the convergence speed, but it may lead to problems such as low training precision and large prediction error. A complex structure can improve the training precision, but it can easily lead to non-convergence or over-fitting. In engineering practice, experience or trial-and-error method are often used in traditional ANN structure design [2,24]. However, because a DBN is deep, with numerous nodes and a complex structure, it is difficult to find the optimal structure using these methods, and the network performance can-not be guaranteed. In addition, these approaches do not result in a network that can self-adapt, which is needed to redesign it for different data sets.

Given the above problems, some researchers have studied DBN structure design. In terms of network depth, Pan et al. proposed using the correlation inference of network energy, network performance, and depth [15]. Gao et al. determined the number of DBN layers using the correlation of hidden layers [16]. But them only analyzed the depth with ignoring the relationship between the number of nodes and the number of layers. Stathakis designed a fitness function to solve the optimal network structure by combining the coding and optimization process of genetic algorithm [18]. However, it is not suitable for the process of unsupervised training. In terms of the number of hidden-layer neurons, researchers have proposed various strategies such as using the data dimensionality as the number of nodes [21], using more nodes than the data dimensionality [20], the minimizing the error to determine the node number, and using a symmetric hidden layer structure [21].

Previous studies have preliminarily discussed the design method for a DBN structure, but they have only discussed a single aspect of structure, either network depth or the number of nodes. Or, they did not fully consider the unsupervised training process of the DBN network. In fact, the performance of a network is determined by both aspects. The two parameters are coupled and hence influence each other. The optimal value of the depth is related to the node selection strategy, and the optimal value of the number of nodes is related to the depth optimization strategy. If we combine the depth decision and the node optimization processes while ignoring the organic correlation between them, it is difficult to obtain a good network structure. Therefore, to improve the performance of DBN by changing its structure, we need a DBN structure design algorithm that simultaneously and organically combines network depth and node number.

Hence, this paper proposes a DBN structural design algorithm based on information entropy and reconstruction error. The algorithm innovatively combines the network depth and number of nodes into a unified mathematical model, introduces information entropy and reconstruction error, and uses the ISA algorithm to solve the optimization problem. First, using information compression and the distribution characteristics of the sample, a bound on the number of hidden layer neurons based on information entropy is derived. In addition, the positive correlation between reconstruction error and network energy is proved, and a model optimization that minimizes the reconstruction error is constructed. Then, this paper employs the ISA algorithm to solve for the network depth and node number while training the network. The experimental results show that this algorithm can generate a network structure that is adapted to different datasets. Moreover, the constructed DBN has lower reconstruction and root-mean-square errors in training process as well as a low prediction error rate in test process.

## 2. Structure Optimization Model of a DBN 

The DBN structure is determined by the number of layers and the number of nodes (or neurons) contained in each layer. Therefore, to adjust the structure, it is essential to automatically solve for the optimal number of layers and nodes for each data set. From the perspective of mathematical modeling, this problem can be expressed as an optimization in the solution space formed by all feasible DBN structures. Therefore, for the general optimization model, the problem can be mathematically expressed in the framework of an objective function and constraint conditions as follows:

(1) $$\begin{array}{ll} \min & f(x)\;\;x\in Χ\\ s.t. & g_{i}(x)=0\;\;i=1,2,\ldots \\ & h_{j}(x)\leq 0\;\;j=1,2,\ldots \end{array}$$

where, f(x) denotes the target function and $g_{i}(x)$ and $h_{j}(x)$ denote equality constraints and inequality constraints, respectively. For the problem of DBN structure design, this paper derives and proves two conclusions:

**Conclusion** **1.** *The range of the number of hidden-layer neurons is based on the information entropy.*

**Conclusion** **2.** *The network performance is based on reconstruction error.*

Hence, the DBN structure optimization model is constructed as follows:

(2) $$\begin{array}{ll} \min & R(C)\;\;C\in \mathbb{C}\\ s.t. & N_{\min}\left(k\right)\leq N_{\mathrm{hid}}(k)\leq N_{\max}\left(k\right),\forall k\in 1\ldots n\\ & D\leq D_{\max} \end{array}$$

Here, *C* represents the DBN structure and ℂ represents the solution space formed by all feasible DBN structures, R(C) indicates the DBN reconstruction error in structure *C*, *k* represents the index of the restricted Boltzmann machine (RBM) in the DBN from 1 to n, $N_{\mathrm{hid}}(k)$ denotes the number of hidden layer neurons in the *k*-th RBM, and $N_{\min}\left(k\right)$ and $N_{\max}\left(k\right)$ represent the minimum and maximum values of the number of neurons in the hidden layer in the *k*-th RBM, respectively. Finally, *D* represents the depth of the DBN network and $D_{\max}$ represents the maximum depth of the network that meets the requirements. The physical meaning of the mathematical model is to find the network structure that minimizes the reconstruction error on the basis of satisfying the boundary for the number of neurons and the upper bound of the depth of network. Section 2.1 and Section 2.2 of this paper detail the derivation of Conclusions 1 and 2, respectively.

### 2.1. Lower Bound of the Number of Hidden Neurons

**Figure 1:**
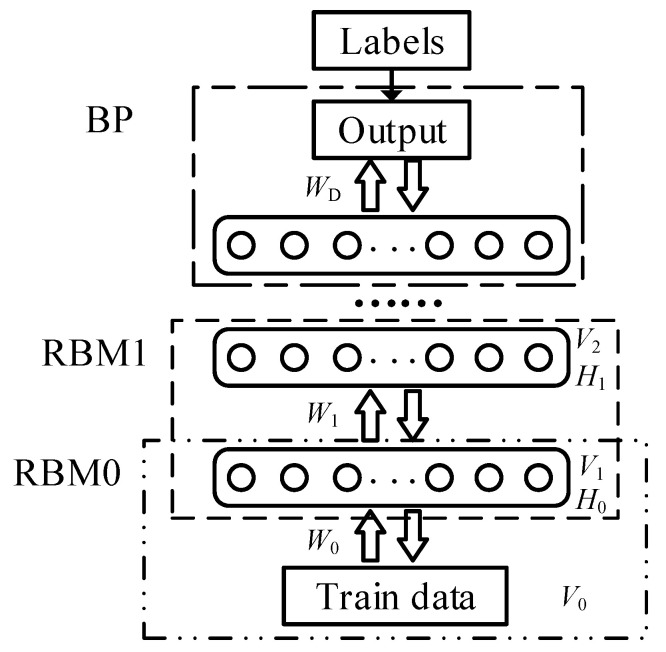
RBM structure in a DBN.

The DBN consists of multiple layers of neurons, where each two adjacent layers of neurons make up one RBM, as shown in Figure 1. Each RBM has a bipartite graph structure. According to the input and output, the neurons are divided into a visible layer and hidden layer. Each neuron only performs layer interconnection and does not perform intra-layer interconnection. Each layer of neurons can be used as both a hidden layer for the current RBM and a visible layer for the next RBM. Therefore, a DBN can be regarded as a deep network in which multiple RBMs are stacked.

The process of transferring data from the visible layer to the hidden layer in an RBM is a dimensionality-reducing feature extraction process [25]. Its purpose is to represent high-dimensional input data using a low-dimensional output vector through network mapping. This feature extraction process, from the viewpoint of information theory, is an information compression process: eliminating the redundant information in the input and using a smaller number of coded bits to achieve the storage of information.

Based on the idea of information compression, when determining the number of hidden-layer nodes, it must be ensured that the maximum amount of information that the hidden layer output vector can store is greater than or equal to the amount of information carried by the input data of the visible layer, so that information will be transferred losslessly. Otherwise, information will be inevitably lost, and this will ultimately reduce the overall network performance. Therefore, this paper employs the information entropy as the criterion for determining the number of hidden layer nodes.

Information entropy, proposed by Shannon, is a measure of information quantity. In physical sense, it refers to the uncertainty of the received signal. The formula for calculating the information entropy of a single character is:

(3) $$H=\sum_{i=1}^{J}p\left(i\right)\log\frac{1}{p\left(i\right)}$$

where, *H* is information entropy, *J* is the number of characters, and p(i) indicates the probability of receiving character *i*, where $\sum_{i=1}^{J}p\left(i\right)=1$.

Equation (3) shows that a larger signal uncertainty leads to a larger amount of information. Moreover, when all the probability values are equal, the amount of information of the character is maximized.

Let the number of visual layer nodes be $N_{\mathrm{viso}}$, the probability that the state of the *i*-th node in a layer equals zero be denoted by $P_{i}\left(0\right)$, and the probability that the state is equal to one be denoted by $P_{i}\left(1\right)$. Then, the information entropy $H_{\mathrm{viso}}$ of the RBM visual layer is calculated by:

(4) $$H_{\mathrm{viso}}=\sum_{i=1}^{N_{\mathrm{viso}}}\left[p_{i}\left(0\right)\log\frac{1}{p_{i}\left(0\right)}+p_{i}\left(1\right)\log\frac{1}{p_{i}\left(1\right)}\right]$$

Further, let the number of hidden layer nodes be $N_{\mathrm{hid}}$, the probability that the state of the *i*-th node in the layer equals zero be denoted by $p_{i}^{\prime }\left(0\right)$, the probability that the state is equal to one be denoted by $p_{i}^{\prime }\left(1\right)$, and the hidden layer’s overall information volume be denoted by $H_{hid}$. Because the state of the hidden layer neurons of DBN can only be zero or one, so the maximum value $H_{\mathrm{hid}}^{\max}$ of $H_{\mathrm{hid}}$ is reached at $p_{i}^{\prime }\left(0\right)=p_{i}^{\prime }\left(1\right)=\frac{1}{2}$:

(5) $$\begin{array}{ll} H_{\mathrm{hid}}^{\max} & =\sum_{i}^{N_{\mathrm{hid}}}-p_{i}^{\prime }(0)\log_{2}\left(p_{i}^{\prime }(0)\right)-p_{i}^{\prime }(1)\log_{2}\left(p_{i}^{\prime }(1)\right)\\ & =\sum_{i}^{N_{\mathrm{hid}}}\frac{1}{2}\log_{2}\left(\frac{1}{2}\right)+\frac{1}{2}\log_{2}\left(\frac{1}{2}\right)=N_{\mathrm{hid}} \end{array}$$

Because the maximum amount of information that the hidden layer output vector can store is greater than or equal to the amount of information carried by the input data of the visible layer, we obtain:

(6) $$H_{\mathrm{hid}}^{\max}\geq H_{\mathrm{viso}}.$$

From Equations (5) and (6), we can get:

(7) $$N_{\mathrm{hid}}\geq H_{\mathrm{viso}}.$$

Obviously, Equation (7) gives the lower bound of the number of nodes in the hidden layer as follows:

(8) $$N_{\min}\left(k\right)=H_{\mathrm{viso}}\left(k\right).$$

To obtain a more reasonable network, the maximum number of neurons in each hidden layer is defined according to [4,21], which use the same number of neurons for the hidden layers. This paper sets the number of nodes for each hidden layer to be no greater than the number of nodes in the input layer. Let $N_{i}$ be the number of nodes in the current layer, and $N_{0}$ be the number of nodes in the input layer. The value range of the number of nodes is as follows:

(9) $$N_{hid}\leq N_{0}$$

From Equation (9), the upper bound of the hidden layer nodes can be obtained as:

(10) $$N_{\max}\left(k\right)=N_{0}$$

From the above analysis, we hence obtain Conclusion 1, and the range of the number of hidden layer nodes based on information entropy is $H_{\mathrm{viso}}\leq N_{hid}\leq N_{0}$.

### 2.2. DBN Performance Measurement Based on Reconstruction Error

To optimize the network structure, we need to introduce an index that can reflect the performance of DBN. According to [20], we have the following lemma:

**Lemma** **1.** *Network energy is an important index for judging the performance of feedback network, and its numerical value is inversely proportional to the network performance.*

Network energy is calculated as:

(11) $$L=\frac{1}{T}\sum_{t=1}^{T}\left[\sum_{i}^{N_{viso}}\sum_{j}^{N_{hid}}W_{ij}v_{i}\left(t\right)h_{j}\left(t\right)+\sum_{i}^{N_{viso}}a_{i}v_{i}\left(t\right)+\sum_{j}^{N_{hid}}b_{j}h_{j}\left(t\right)\right]$$

Here, *L* represents the network energy, *T* represents the total number of training samples, *W* represents the weight matrix, $v_{i}(t)$ represents the value of the *i*-th visible-layer neurons, $h_{j}(t)$ represents the value of the *j*-th hidden-layer neurons, $a_{i}$ represents the bias of the *i*-th visible-layer neurons, and $b_{j}$ represents the bias of the *j*-th hidden-layer neurons. A lower network energy indicates a better network performance.

Therefore, in theory, network energy can be used as an optimization objective. However, the computational complexity of network energy is high, which may lead to impractically long computation times and memory overflow. Hence, in this paper, based on [15], the relationship between reconstruction error and network energy is derived, and a network performance metric based on reconstruction error is proposed.

The reconstruction error refers to the difference between the samples obtained by Gibbs sampling and the original data. The calculation of reconstruction error *R* is:

(12) $$R=\frac{\sum_{t=1}^{T}\tilde{v}(t)-{\tilde{v}}_{0}(t)}{T}$$

Here, ${\tilde{v}}_{0}(t)$ denotes the original data and $\tilde{v}(t)$ denotes the value obtained by Gibbs sampling. Because the input of samples is stationary processes, when *T* is large enough:

(13) $$\frac{\sum_{t=1}^{T}\tilde{v}\left(t\right)}{T}=E\left(\tilde{v}\right)=\sum_{k}p_{v}\left(k\right)k$$

(14) $$\frac{\sum_{t=1}^{T}{\tilde{v}}_{o}\left(t\right)}{T}=E\left({\tilde{v}}_{o}\right)=\sum_{k}p_{v_{0}}\left(k\right)k$$

Here, Ε(•) denotes the expectation, $p_{v}(k)$ denotes the probability that reconstruction value $\tilde{v}$ equals *k* (this is also called posteriori probability), and $p_{v_{0}}(k)$ as the probability that reconstruction value ${\tilde{v}}_{0}$ equals k (this is also called priori probability). Combining Equations (12)–(14), we get:

(15) $$R=\sum_{k}k\left[p_{v}\left(k\right)-p_{v_{0}}\left(k\right)\right]$$

In RBMs, we use $v_{0}$ to denote the original data of the visible layer, v to denote the value after reconstruction, and *h* to denote the value of hidden layer. For convenience of discussion, the probability distribution of v is p(v), the probability distribution of $v_{0}$ is $p\left(v_{0}\right)$, and the probability distribution of *h* is p(h). According to conditional probability and total probability formula, p(v) is calculated as follows:

(16) $$\begin{array}{ll} p\left(v\right) & =\sum_{h}p\left(v|h\right)p\left(h\right)\\ & =\sum_{h}p\left(v|h\right)\sum_{v_{0}}p\left(h|v_{0}\right)p\left(v_{0}\right)\\ & =\;\sum_{h}\sum_{v_{0}}\frac{p\left(v,h\right)}{p\left(h\right)}\frac{p\left(h,v_{0}\right)}{p\left(v_{0}\right)}p\left(v_{0}\right)\\ & =\;\sum_{h}\sum_{v_{0}}p\left(v,h\right)\frac{p\left(v_{0},h\right)}{p\left(h\right)}=\sum_{h}\sum_{v_{0}}p\left(v,h\right)p\left(v_{0}|h\right) \end{array}$$

Because $p\left(v_{0}\right)$ belongs to priori probability, $p\left(v_{0}|h\right)=p\left(v_{0}\right)$. Equation (15) can be rewritten as follows:

(17) $$R=\sum_{k}k\left[\sum_{h}\sum_{v_{0}}p_{v,h}\left(k,h\right)p_{v_{0}}\left(k\right)-p_{v_{0}}\left(k\right)\right]$$

Because $p_{v_{0}}\left(k\right)$ is only related to the training data and has nothing to do with the network, the following statement can be obtained from Equation (17):

(18) $$R\propto p_{v,h}\left(k,h\right)$$

Combining Equation (11) and the energy-based model of RBM, $p_{v,h}\left(k,h\right)$ has the following relationship with network energy *L*:

(19) $$p_{v,h}\left(k,h\right)=\frac{e^{L}}{Z}$$

Here Z is a normalized denominator that is determined only by the network parameters. Therefore, according to Equation (19), we obtain:

(20) $$p_{v,h}\left(k,h\right)\propto L$$

Moreover, according to Equations (18) and (19), we have:

(21) $$R\propto p_{v,h}\left(k,h\right)\propto L$$

This demonstrates that the reconstruction error has a positive correlation with the network energy. The computational complexity of Equations (11) and (17) is shown in Table 1. Obviously, the computational complexity of the reconstruction error is much lower than that of the network energy. Therefore, according to Equation (21), we obtain Conclusion 2.

## 3. Structure Design Using ISA

For the optimization model established in the Section 2, a suitable algorithm can be adopted. The simulated annealing (SA) algorithm has many advantages [26], such as a simple structure, flexibility, and high efficiency. At the same time, the simulated annealing algorithm has been theoretically proved to be a global optimization algorithm [27]. Moreover, the network performance oscillation caused by the DBN structure optimization process is similar to the “heating” and “cooling” procedure of the SA algorithm, so this algorithm is easily incorporated into DBN structure design. Hence, this section explains how we employ the SA algorithm to optimize the mathematical model described in Section 2.

The SA algorithm is a general probabilistic search algorithm that simulates the annealing process of solid matter in physics. It has a fast search speed and excellent globally optimal search ability. The core concept of SA is to construct a state transition probability matrix and update the current solution according to the matrix. The probability of a transition from state 1 to state 2 p(1→2) is:

(22) $$p\left(1\to 2\right)=\{\begin{array}{cc} 1, & Y_{2}<Y_{1}\\ \exp\left(-\frac{Y_{2}-Y_{1}}{\tau }\right), & Y_{2}>Y_{1} \end{array}$$

Here, τ is the “temperature”, which is the artificially set control algorithm iteration rate, $Y_{1}$ and $Y_{2}$ are the internal energies of state 1 and 2, respectively, and the state energy *Y* is the optimization objective.

In addition, let τ be gradually reduced in each iteration according to:

(23) $$\tau_{k+1}=\alpha \tau_{k}$$

Here, α denotes the descending factor, α<1, to ensure τ decreases. Obviously, combining Equations (22) and (23), as the temperature τ gradually decreases, the system state will gradually converge to a low energy state and eventually reach the lowest point of the internal energy, that is, the minimum value of the optimization target.

The traditional SA algorithm has some disadvantages, such as sensitive parameters, poor convergence performance, and a tendency to fall into local optima. Therefore, according to [27], the global search performance of SA can be improved by adding memory and return search functions. The improved algorithm is called the ISA algorithm.

In order to study the DBN structure design based on ISA algorithm, two lemmas are introduced.

**Lemma** **2.** *the fitting accuracy of the network increases as the number of network layers increases, when the number of training samples is sufficient [15]*.

**Lemma** **3.** *increasing network depth can improve network performance more effectively than increasing network width [28]*.

Combining Conclusions 1 and 2, we obtain the following three Rules.

1. The internal energy of the solution in the ISA algorithm is equal to the reconstruction error of the RBM at the highest level of the DBN.

From Conclusion 2 and Lemma 2, we obtain that the reconstruction error of the topmost RBM reflects the upper bound of the performance of the whole network structure, which is the optimization goal of the model. Hence, we obtain a second rule.

2. The undetermined new solution of the number of nodes in the layer is randomly generated, and the state update follows Equation (22).

The number of nodes $N_{i}$ in the layer is randomly generated from the average probability distribution, where the probability of each value is $P=\frac{1}{M}$ and M is the total number of possible values. Based on Conclusion 1 and Equation (8), the number of neuron nodes in the current layer $N_{i}$ and the number of nodes in the next layer $N_{i-1}$ have the following relationship:

(24) $$N_{0}\geq N_{i}\geq \log_{2}\left(N_{i-1}\right)$$

Hence, we obtain the following equation:

(25) $$M=N_{0}-\mathrm{ceil}\left(\log_{2}\left(N_{i-1}\right)\right)$$

According to the Metropolis rules, if $N_{i}^{\prime }$ denotes the undetermined new solution, then the probability of accepting state update $N_{i}\to N_{i}^{\prime }$ is calculated according to Equation (22), where the reconstruction error $Y_{2}$ under $N_{i}^{\prime }$ is substituted into $R_{i}^{\prime }$ and the reconstruction error $Y_{1}$ under $N_{i}$ is replaced by $R_{i}$. We finally have a third rule.

3. The number of layers increases monotonically from simple to complex.

According to Lemma 3, the effect of the upper layer nodes on performance is much higher than that of the lower layer nodes, so the complexity of the network structure is gradually improved by a layer-by-layer approach. The number of nodes in the bottom layer is optimized first then fixed. Then, in each subsequent iteration, only the number of nodes in the next layer of the network is adjusted.

The pseudocode of the resulting DBN structure design algorithm is shown in Algorithm 1.
**Algorithm 1: DBN Structure Design Algorithm via ISA**1:Initialization: set initial temperature *τ*_0_, minimum temperature *τ*_min_, intra-layer iteration limit *D*_max_, network overall iteration limit *G*_max_, objective function threshold *R_end_*, initial network depth *D* = 2 (input layer and output layer), and memory matrix *I*.2:**For***i* = 1: *D*_max_ align all the symbols correctly3:    *D* = *D* + 1, *T* = *T*_0_4:    Generate *N_i_* from Rule 2, form current network structure *C* based on *N_i_*, and calculate the reconstruction error *R* of *C*.5:    **For** *j* = 1: *G*_max_6:        The new number of neurons *N*′ is randomly generated by Rule 2 as the undetermined solution, the DBN structure *C*′ formed by *N*′ is the candidate DBN structure, and the reconstruction error *R*′ corresponding to *C*′ is calculated.7:        **If** Δ*R* = *R*′ − *R* < 0 or exp(−Δ*R*/*T*) > *rand*8:            C = *C*′, *j* = 1: *G*_max_9:        **If** j ≥ *G*_max_^1^ or *T* ≤ *T*_min_ or R ≤ *R_end_*10:            Find *C_best_* in *I* and search the adjacent domain of *C_best_* to obtain *Cfinal*, then go to Step 3.11:            *τ_k_*_+1_ = *ατ_k_*12:    **End For**13:    **If** D ≥ *D*_max_ or R ≤ *R_end_*14:        Return the optimal network structure.15:**End For**

## 4. Experiments and Results Analysis

In the evaluation, we refer to the proposed algorithm as the information entropy and reconstruction error via ISA (IEREISA) method. We compare the similarities and differences in performance between IEREISA and some common DBN depth and node-number setting methods. The depth setting methods consist of a fixed method [25], a depth design method based on the reconstruction error [15], and a depth design method based on the number of correlations [16]. The node setting methods consist of using a fixed number of nodes [15], and an error minimization method [25]. Combining these methods, we obtain three comparison algorithms. Moreover, to evaluate the effect of the ISA algorithm in IEREISA, a DBN structure design algorithm SA is also compared. The comparison algorithms are as follows:

- Reconstruction Error and Equivalent nodes (REE): The number of neurons in each layer are set to be equal and the decision to increase the network depth is determined by the value of the reconstruction error. Moreover, the maximum network depth is set to ensure the convergence of the algorithm.
- Rate of Correlation and Equivalent nodes (RCE): Similar to REE, the numbers of neurons in each layer are equal. The value of the cross-correlation coefficient determines whether to increase the network depth and the maximum network depth is fixed to ensure the convergence of the algorithm.
- Traversal Search with Constant Layers (TSCL): TSCL obtains the optimal architecture by manually setting the network depth and then searching for the number of neurons in each layer by traversal, also called exhaustive search. In the TSCL algorithm, the maximum number of neurons per layer is fixed to ensure the convergence of the algorithm.
- IERESA: The main idea of the IERESA algorithm is the same as the IEREISA algorithm, except that the normal SA algorithm is used instead of ISA.

The corresponding DBNs were generated for the above five different structural algorithms, and experiments were carried out on three public datasets (Cifar-10, Cifar-100, and MNIST) [29]. The results consist of the following four metrics:

1. Reconstruction error in the unsupervised training process. The unsupervised training pre-adjusts the weights and bias, and a lower reconstruction error indicates better training, which further indicates that the structure design algorithm obtains better results.
2. Root-mean-square error (RMSE) in the supervised training process. Supervised training uses the error back propagation algorithm to fine-tune the weight. A lower RMSE after training indicates better training and a better network performance.
3. The prediction error rate of the test dataset. The error rate of the test results indicates the effectiveness of the algorithm.
4. The runtime of the algorithm. When the DBN structure is changed, the new part of the structure needs to be retrained, which causes the complexity of the algorithm to substantially impact training time. A higher complexity and larger number of required iterations increases the time for training. Therefore, runtime, as an indicator of algorithm complexity, can be compared across different algorithms.

In the experiment, the initialization parameters of the DBN network were set as follows:

(1) The weights *W* were randomly generated according to the normal distribution *N* ~ (0, 0.01).
(2) The hidden layer bias *c* was initialized to be zero.
(3) To control the network scale, *D*_max_ = 10.
(4) The visual layer bias *b* was produced by the following equation:

(25) $$M=N_{0}-\mathrm{ceil}\left(\log_{2}\left(N_{i-1}\right)\right)$$

where *b_i_* is the bias of the *i*-th neuron and *p_i_* is the probability that the neuron will become active. The remaining DBN initialization parameters are controlled by the input dataset. The DBN initialization parameters for each specific experiment are listed in Table 2 and Table 3 below.

### 4.1. Cifar-10 Dataset Classification Experiment

This experiment tests the performance of the methods on a high-dimensional input sample. The public dataset Cifar-10 is a classic experimental dataset in the machine learning, which has 60,000 samples and 10 classes. Each sample contains features and labels, characterized by 3072 pixels with a value of 1–255 and a single integer in the range 0–9. We used 50,000 samples as training set and 10,000 samples the test set, and the algorithm parameter settings are shown in Table 2. In the IEREISA and IERESA algorithms, *R_end_* = 1. In the REE and RCE algorithms, the number of neurons in each layer was 200 and 100, and in the TSCL algorithm, the number of hidden layers in the network was 10.

#### 4.1.1. Reconstruction Error for Unsupervised Training 

**Figure 2:**
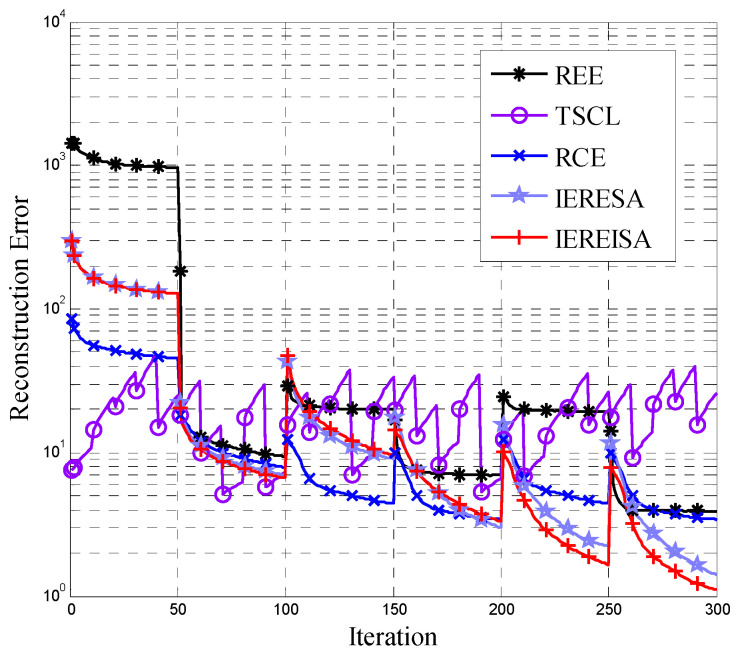
DBN reconstruction error variation of five structural algorithms on the Cifar-10 dataset.

The reconstruction error for DBN obtained by the five structure design algorithms is shown in Figure 2. Obviously, over the whole iteration process, except for the TSCL algorithm, the reconstruction error of the algorithms gradually decreases. The IEREISA algorithm has the lowest convergence value, demonstrating that it performs the best on this dataset.

In Figure 2, the REE algorithm and the RCE algorithm use an equal number of neurons in each layer, which does not guarantee that the numbers of neurons in each layer are optimal. Hence, the reconstruction error cannot converge to its optimal value. It proves that the performance of DBN is determined by the number of layers and the number of nodes. The algorithms that only consider the number of layers cannot find the optimal network structure. Moreover, the TSCL algorithm adopts the traversal method with a slow convergence speed, so the reconstruction error tends to oscillate and may not converge within the maximum number of iterations. In the same way, an algorithm that considers only the number of nodes without considering the number of layers also cannot find the optimal network structure. In addition, the IEREISA algorithm and IERESA algorithm have good performance and the IEREISA algorithm can reach the lowest reconstruction error. This is because the optimization ability of SA is not as good as that of ISA. The experimental results hence show that the network structure generated by IEREISA algorithm has the lowest reconstruction error and the IEREISA algorithm, which simultaneously and organically combines network depth and node number, can find the optimal DBN structure suitable for the current dataset.

The DBN structures obtained by the above five algorithms is shown in Table 3. It can be seen that the DBN structure obtained by the IEREISA algorithm proposed in this paper is more reasonable than other algorithms.

#### 4.1.2. RMSE in Supervised Training

**Figure 3:**
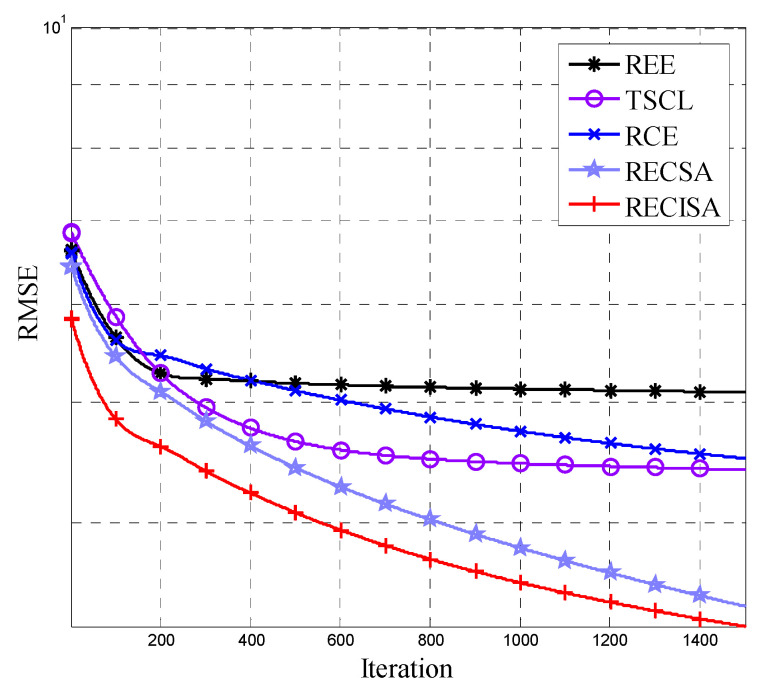
RMSE variation of five algorithms on the Cifar-10 dataset.

The algorithm parameter settings for the supervised training process are shown in Table 2. The RMSE of the DBN networks generated by the algorithms during the training process is shown in Figure 3. Compared with the other four algorithms, the DBN network generated by the IEREISA algorithm has the fastest convergence speed for supervised training and has the lowest RMSE convergence value, because the IEREISA can design the most proper network structure.

#### 4.1.3. Prediction Error Rate and Time Complexity

**Figure 4:**
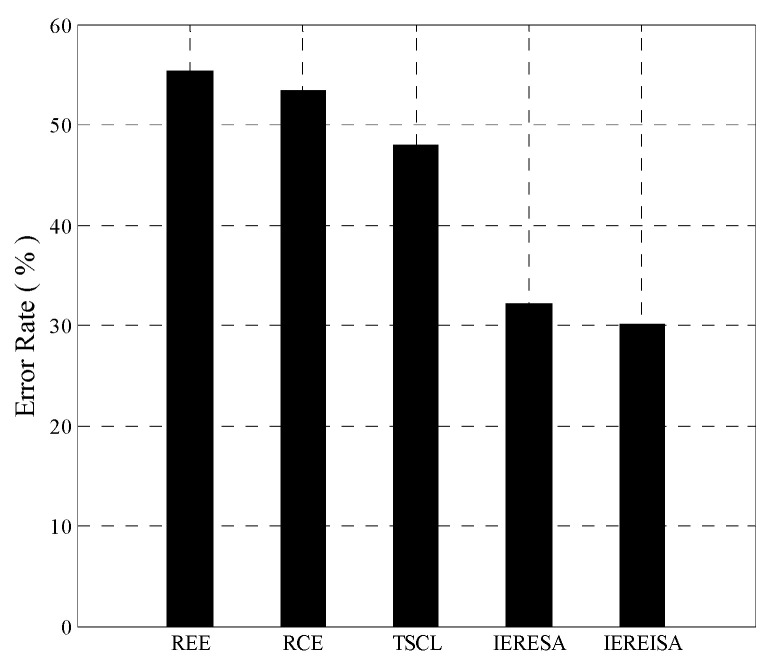
Prediction error rate of five algorithms on the Cifar-10 dataset.

**Figure 5:**
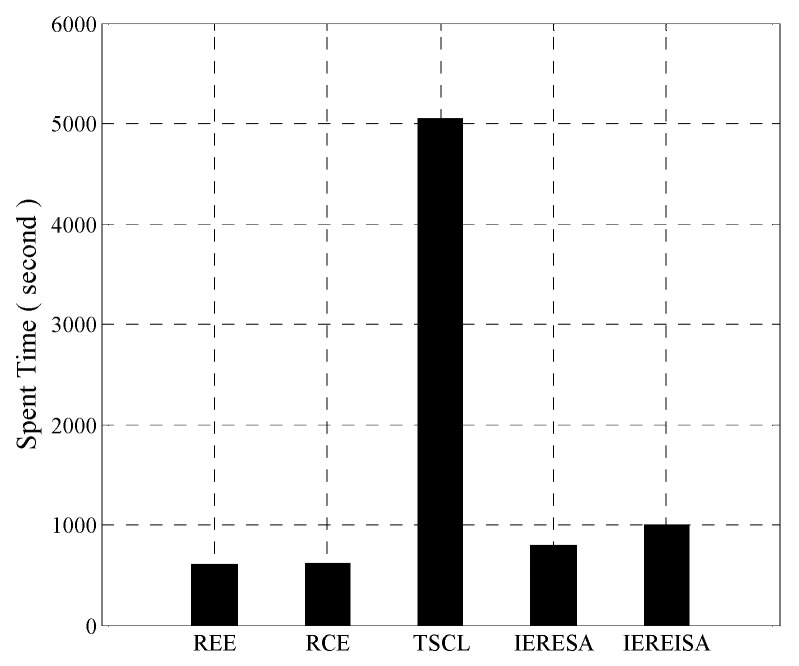
Runtime of five algorithms on the Cifar-10 dataset.

The trained networks were tested using the same test set, and the error rates are shown in Figure 4. The IEREISA algorithm has the lowest error rate of 30.35%. The runtime statistics of the algorithms are shown in Figure 5. The training times of RCE and REE algorithms are short, the training times of the IERESA and IEREISA algorithms are a little longer, and the training time of the TSCL algorithm is the longest. This is because the number of nodes is much larger than the number of layers of the solution space, so the IERESA, IEREISA, and TSCL algorithms require more searching and take a longer time to compute. In particular, the TSCL algorithm uses traversal search, which is inefficient. Although the IEREISA algorithm takes more time than some methods, it considers both the network depth and number of nodes. In contrast to the REE and RCE algorithms, IEREISA obtains both the best network depth and the number of nodes. IEREISA also improves the quality of the solution obtained by IERESA.

In summary, the experimental results show that on the Cifar-10 dataset, the proposed IEREISA algorithm can obtain a lower RMSE and reconstruction error than those of other algorithms and has higher prediction accuracy. However, the algorithm incurs a small increase in time complexity owing to the increased scale of the solution space.

### 4.2. MNIST Dataset Classification Experiment

This experiment evaluates the performance of the algorithm on other datasets. The experiment uses the MNIST handwriting recognition dataset, which is a basic experimental dataset for testing network performance and consists of a total of 60,000 training samples, 10,000 test samples and 10 classes. Each sample has a 28 × 28 matrix as the input features and 10 one-hot vectors as labels. The algorithm parameters were set as shown in Table 4.

In the IEREISA and IERESA algorithms, *R_end_* = 1. In the REE and RCE algorithms, the number of neurons in each layer was 200, and in the TSCL algorithm, the number of hidden layers in the network was 10.

#### 4.2.1. Reconstruction Error in Unsupervised Training 

**Figure 6:**
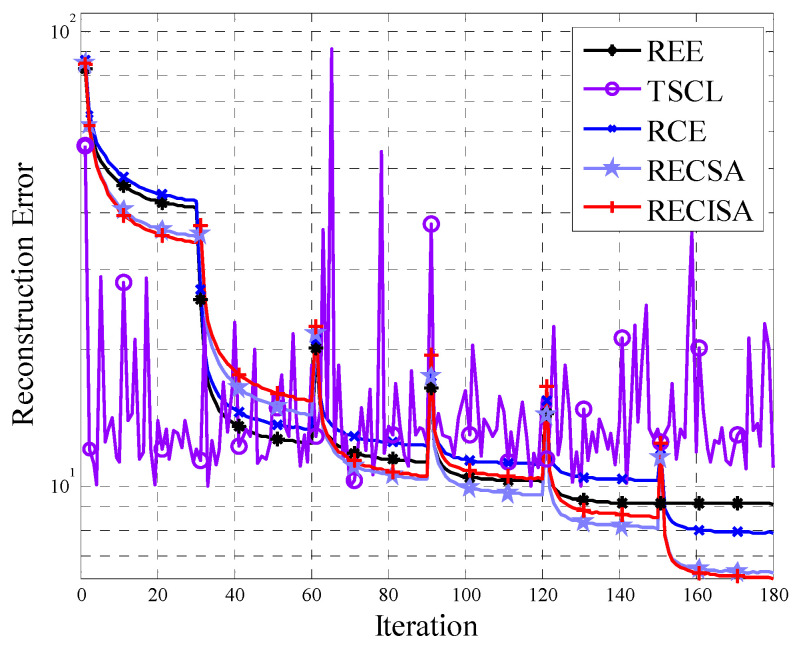
DBN reconstruction error variation of five algorithms on the MNIST dataset.

The results of the reconstruction error are shown in Figure 6. Like the analysis in Section 4.1.1, the IEREISA algorithm also achieves the lowest reconstruction error on the MNIST dataset, which demonstrates the effectiveness of the algorithm on more than one dataset.

The DBN structures obtained by the above five algorithms is shown in Table 5. It has also been proved in Table 5 that the IEREISA algorithm proposed in this paper has the most reasonable network structure, which shows the same result as in Table 3.

#### 4.2.2. RMSE in Supervised Training 

**Figure 7:**
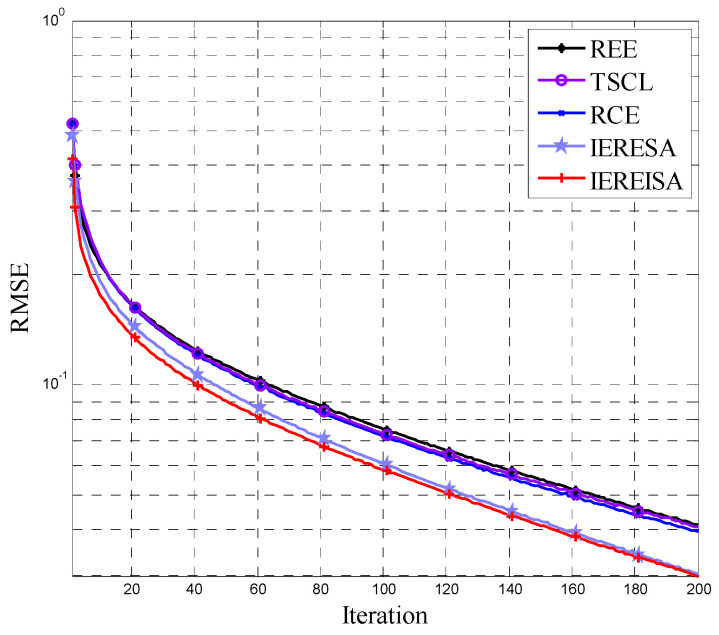
RMSE variation of DBN of five algorithms on the MNIST dataset.

The results of the RMSE are shown in Figure 7. The RMSE of the IEREISA algorithm converges to the lowest value and its speed of convergence is the fastest on the MNIST data set. Compared with the networks of the other algorithms, the DBN structure designed by the proposed IEREISA algorithm has the most proper structure and shows the best fitting ability.

#### 4.2.3. Prediction Error Rate and Time Complexity

**Figure 8:**
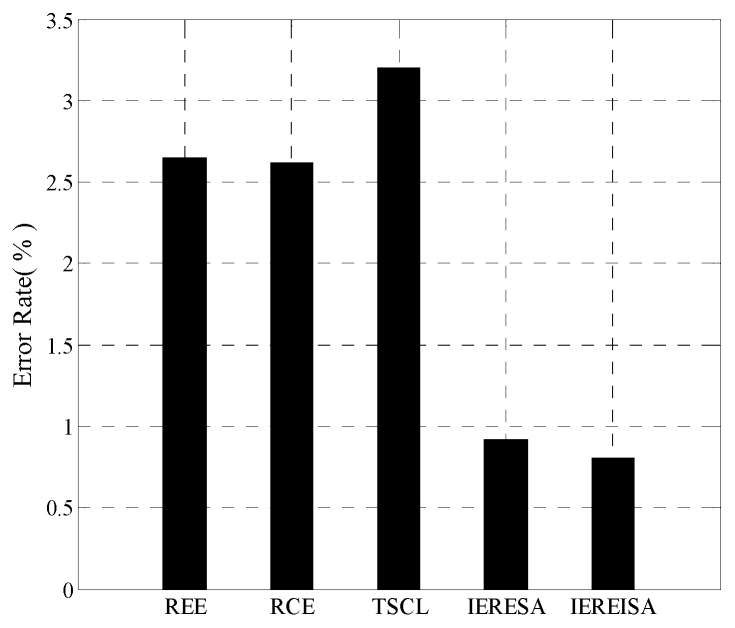
Prediction error rate of five algorithms on the MNIST dataset.

The error rates are compared shown in Figure 8. The error rate of the IEREISA algorithm (0.81%) is much lower than of the other four algorithms. This demonstrates that the network structure generated by the IEREISA algorithm has the best prediction performance on the MNIST dataset compared with other algorithms.

**Figure 9:**
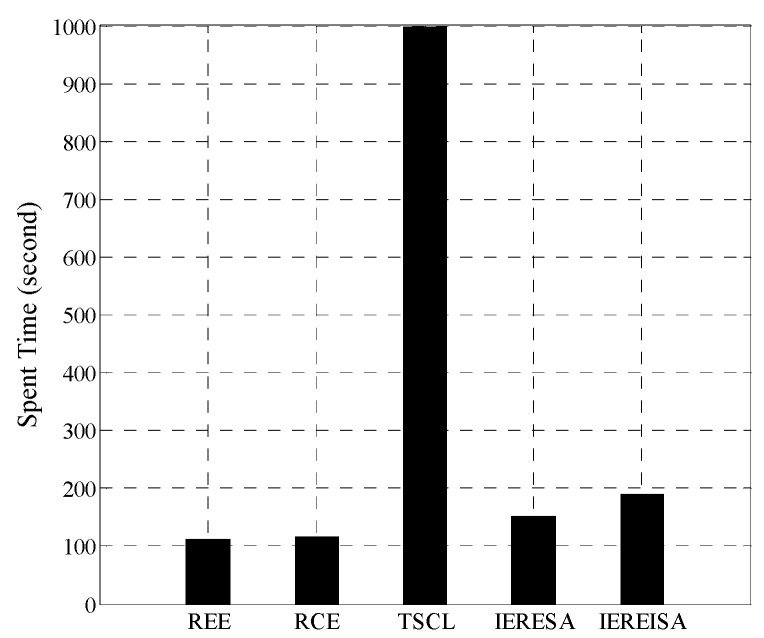
Runtime of five algorithms on the MNIST dataset.

The time consumed by the five algorithms is shown in Figure 9. The IEREISA algorithm slightly increases the time complexity of the algorithm, which is consistent with the experimental results of Section 4.1.3.

### 4.3. ISA Algorithm Analysis

In the DBN structure design algorithm proposed in this paper, when the RBM layer is newly added, the ISA algorithm is selected to calculate the optimal number of neurons. In order to verify the effectiveness of the ISA algorithm, the ISA algorithm is compared with the SA algorithm and the genetic algorithm (GA). The experiment using genetic algorithm was denoted as IEREGA. In the experiment, the parameter settings of the IEREISA algorithm and the IERESA algorithm are shown in Table 2 and Table 3. The parameter settings on Cifar-10 dataset are same as Cifar-100 dataset. According to [18], the parameter settings of IEREGA algorithm are as shown in Table 6.

The experimental results of three algorithms on the three datasets are shown in Table 7, Table 8 and Table 9. By comparing Table 7, Table 8 and Table 9, it can be seen that the IEREISA algorithm can obtain a reasonable network structure for different datasets while maintaining low reconstruction error, low RMSE, and high prediction accuracy. Table 8 shows that the SA algorithm may fall into local optima when solving for the number of neurons, which is caused by the SA algorithm’s performance.

It can be seen from Table 9 that the IEREGA algorithm also appears to fall into the local optimum, because GA is susceptible to the initial value of the population. When searching the optimal number of neurons, the area of solutions determined by the coding length of GA is much larger than the range of values satisfying the constraints of neurons, thus causing a decline in GA search capability. And the quality of the solution is affected by the insufficient local search ability of GA. 

In summary, for different datasets, the proposed IEREISA algorithm maintains the lowest reconstruction error, RMSE and prediction error rate, and has the best fitting and prediction performance compared with other algorithms. The IEREISA algorithm organically combines the methods for determining the number of layers and number of neurons, and simultaneously optimizes both to obtain a better network structure. Compared with the REE and RCE algorithms which only consider the number of layers, the runtime of IEREISA algorithm is longer, but redundancy in the network is avoided. Moreover, a network with better performance and a more reasonable structure is obtained by the IEREISA algorithm. Compared with TSCL, which only considers the number of neurons, IEREISA can not only obtain a network with better performance, but it also improves the efficiency of the algorithm and reduces the runtime. Because TSCL adopts a traversal search, it is difficult to converge for networks with a complex structure.

Compared with the previously proposed method, the IEREISA algorithm, which utilizes information entropy and reconstruction error, optimizes the number of layers and the number of neurons simultaneously and can quickly obtain a DBN network with better performance and a more reasonable structure.

## 5. Conclusions

In this paper, an approach that combines and simultaneously optimizes the number of network nodes and the depth of the network in a DBN was proposed. First, we constructed a mathematical model for optimizing the DBN structure by introducing information entropy and reconstruction error. Then, the ISA algorithm was employed to optimize the model. Finally, the algorithm proposed in this paper was tested on three public datasets. Experimental results show that for different datasets, the proposed algorithm can achieve lower reconstruction error, RMSE, and prediction error rates. Moreover, this algorithm can adaptively optimize a network structure for different datasets and obtain a better network structure than other algorithms. The DBN structure design algorithm proposed in this paper is superior to the previously proposed algorithms and can be used to provide a reference for the setting of DBN structural parameters for different datasets, which is an important and often over-looked issue of parameter optimization in DBN.

The ideas in this article can also be used when working with other network models. For example, for the CNN model, the reconstruction error after optimization for CNN can be used as an objective function of network performance. The information entropy theory is used as the constraint condition of the number of neurons, and the heuristic search algorithm can be used to obtain the optimal network structure. In this paper, we mainly combine the unsupervised training process of DBN, so the algorithm proposed in this paper may not be applicable to networks without unsupervised training process. Therefore, our follow-up work will be based on the idea of this paper, and propose structure design algorithms for other network models.

## Figures and Tables

**Table 1:** Computational complexity of reconstruction error and network energy.

Means	Multiplication Quantity	Addition Quantity
Reconstruction Error	T(VH+1)	TVH−1
Network Energy	T(2VH+V+H)+1	T(VH+V+H)−1

**Note.***V* and *H* represent the number of neurons in all visible layers and hidden layers, respectively.

**Table 2:** Algorithm parameter settings for the Cifar-10 dataset.

Batch Size	Iterations (Supervised, Unsupervised)	Learning Algorithm	Momentum	Learning Rate (Supervised, Unsupervised)	Activation Function	Output Classifier	*τ*_0_	*α*
2000	(1500,50)	Momentum gradient	0.5	(0.5,0.5)	Sigmoid	Softmax	1	0.7

**Table 3:** The five DBN structures obtained by above five algorithms in Cifar-10 dataset.

Algorithm	DBN Structure	Reconstruction Error
REE	[3072,200,200,200,200,200,200,10]	3.9989
TSCL	[3072,3008,2009,500,507,406,99,208,316,58,36,10]	5.0036
RCE	[3072,100,100,100,100,100,100,10]	3.6587
IERESA	[3072,2959,756,1024,146,99,95,10]	1.4032
IEREISA	[3072,2958,756,1033,134,99,95,10]	1.1106

**Table 4:** Algorithm parameter settings for the MNIST dataset.

Batch Size	Iterations (Supervised, Unsupervised)	Learning Algorithm	Momentum	Learning Rate (Supervised, Unsupervised)	Activation Function	Output Classifier	*T*_0_	*α*
200	(30,500)	Momentum gradient	0.5	(0.5,0.5)	Sigmoid	Softmax	5	0.5

**Table 5:** The five DBN structures obtained by above five algorithms in MNIST dataset.

Algorithm	DBN Structure	Reconstruction Error
REE	[784,200,200,200,200,200,200,10]	3.9989
TSCL	[784,777,659,452,68,106,69,78,16,28,36,10]	5.0036
RCE	[784,100,100,100,100,100,100,10]	3.6587
IERESA	[784,150,138,112,102,92,82,10]	1.4032
IEREISA	[784,155,150,112,112,100,75,10]	1.1106

**Table 6:** Parameter settings of the IEREIGA algorithm on different datasets.

Dataset	Coding Length	Population	Max Number of Generations	Crossover Probability	Mutation Probability
Cifar-10	12	10	10	0.75	0.01
Cifar-100	12	10	10	0.75	0.01
MNIST	10	10	10	0.75	0.01

**Table 7:** Experimental results of the IEREISA algorithm on different dataset.

Dataset	Number of Layers	Number of Neurons	Reconstruction Error	RMSE	Prediction Accuracy
Cifar-10	8	[3072,2958,756,1033,134,99,95,10]	1.1106	3.3010	69.65%
Cifar-100	10	[3072,2586,880,112,86,73,99,95,86,100]	36.2558	10.0777	61.94%
MNIST	8	[784,155,150,112,112,100,75,10]	6.2096	0.0299	99.19%

**Table 8:** Experimental results of the IERESA algorithm on different dataset.

Dataset	Number of Layers	Number of Neurons	Reconstruction Error	RMSE	Prediction Accuracy
Cifar-10	8	[3072,2959,756,1024,146,99,95,10]	1.4032	3.4263	67.43%
Cifar-100	10	[3072,2516,892,117,86,73,98,95,85,100]	36.8585	11.7817	61.70%
MNIST	8	[784,150,138,112,102,92,82,10]	6.2397	0.0302	99.08%

**Table 9:** Experimental results of the IEREGA algorithm on different dataset.

Dataset	Number of Layers	Number of Neurons	Reconstruction Error	RMSE	Prediction Accuracy
Cifar-10	9	[3072,2436,1056,102,461,156,114,95,10]	2.0031	3.4003	64.34%
Cifar-100	10	[3072,2516,892,201,88,98,102,94,85,100]	38.6475	11.8016	61.60%
MNIST	8	[784,155,150,112,107,95,74,10]	6.3305	0.0311	99.07%
